# “Health as Work”, Structural Vulnerability Shaping Migrant Health in Non-Metropolitan Spain: A Qualitative Study

**DOI:** 10.3390/healthcare14193202

**Published:** 2026-09-28

**Authors:** Elena Andina-Díaz, Carmen Villar-Bustos, Enedina Quiroga-Sánchez

**Affiliations:** 1 HeQoL Research Group, CECAVIS Research Group, IbioLeon, Department of Nursing and Physiotherapy, Faculty of Health Sciences, Universidad de León, 24170 León, Spain; elena.andina@unileon.es; 2 EYCC Research Group, University of Alicante, 03690 Alicante, Spain; 3 Zamora University School of Nursing (Affiliated Centre), University of Salamanca, 49022 Zamora, Spain; 4 Department of Nursing and Physiotherapy, Faculty of Health Sciences (Campus de Ponferrada), Universidad de León, 24404 Ponferrada, Spain; equis@unileon.es

**Keywords:** emigration and immigration, qualitative research, structural vulnerability, health equity, cultural competency, nursing, social determinants of health

## Abstract

**Highlights:**

**What are the main findings?**

- Health as Work emerged as the central concept explaining migrants’ health experiences.
- Administrative precarity and occupational insecurity influenced health more than healthcare access alone.

**What are the implications of the main findings?**

- Nurses can reduce structural vulnerability by strengthening cultural competence, facilitating healthcare navigation, and advocating for equitable access to care.
- Integrated health and social policies are needed to improve migrant health in smaller communities.

**Abstract:**

**Background/Objectives**: Migrants often experience health inequities resulting from administrative insecurity, precarious employment, and barriers to healthcare access. Although non-metropolitan areas are increasingly important destinations for migrant populations, evidence on their health experiences in these settings remains limited. This study aimed to examine how migrants living in non-metropolitan areas of Spain experience health throughout the migration process and to identify implications for nursing practice and health policy. **Methods**: A qualitative phenomenological design was used to explore migrants’ lived experiences. Twenty-two adult migrants living in the Spanish provinces of Zamora and León participated in semi-structured, in-depth interviews. Data were analyzed using thematic analysis following Braun and Clarke, and the study adhered to the Consolidated Criteria for Reporting Qualitative Research (COREQ). **Results**: Five interconnected themes emerged: migration process, health perceptions, social networks, work conditions, and adaptation and integration. The central finding, “Health as Work”, revealed that participants primarily understood health as the ability to work and maintain economic stability rather than as a state of physical or mental well-being. This perspective reflected broader structural vulnerability arising from administrative insecurity, precarious employment, and limited social support. Nurses and third-sector organizations were identified as essential in facilitating healthcare navigation, promoting health literacy, and providing psychosocial support. **Conclusions**: Migrants’ health experiences in non-metropolitan Spain are shaped by structural vulnerability, with employment and legal status strongly influencing health and access to care. Nursing practice should integrate culturally responsive and structurally informed care, while health policies should reduce administrative and employment barriers and strengthen collaboration between healthcare services and community organizations to promote health equity.

## 1. Introduction

International migration is reshaping health systems worldwide, requiring healthcare services to respond to increasingly diverse populations while addressing persistent health inequities. Migrants frequently encounter barriers to healthcare, employment, housing, and social participation, resulting in poorer health outcomes and additional demands on health systems [1,2,3]. Despite international commitments to Universal Health Coverage and the Sustainable Development Goals (SDGs), inequities related to administrative insecurity, labor precarity, discrimination, and limited access to healthcare remain common among migrant populations [4,5].

Migrants’ health is strongly influenced by the social determinants of health, including employment, legal status, income, education, and access to healthcare [6]. Administrative insecurity, delayed regularization procedures, precarious employment, and difficulties in recognizing professional qualifications may generate cumulative disadvantages that compromise physical and mental health, social integration, and continuity of care [7,8]. These structural conditions challenge health systems’ capacity to provide equitable care, highlighting the need for interventions that address both healthcare delivery and the broader determinants of health [9].

Nurses are central to this response by facilitating healthcare access, promoting health literacy, coordinating care, and advocating for vulnerable populations. Leininger’s Theory of Culture Care Diversity and Universality [10] and Campinha-Bacote’s model of cultural competence [11] provide complementary frameworks for delivering culturally responsive care. Understanding migrants’ lived experiences is essential for designing person-centered, culturally responsive healthcare services that effectively address structural and social barriers to health [12].

Although migrant health has been widely investigated in metropolitan areas, less is known about the health experiences of migrants in non-metropolitan settings [13,14]. In this study, the term “non-metropolitan” refers to the OECD classification of small regions (TL3), where less than 50% of the population resides in a functional urban area (FUA) of at least 250,000 inhabitants. Non-metropolitan regions are further distinguished based on their access to metropolitan areas, small and medium-sized cities, or remote zones [15]. This distinction is relevant because healthcare resources, employment opportunities, social support networks, and services aimed at migrants can vary depending on the territorial context [14,16]. Recent qualitative evidence from similar settings suggests that migrants’ healthcare experiences are shaped by the interplay of social, cultural, environmental, and institutional factors, while local resources and social relationships can facilitate access, integration, and well-being [17,18,19]. However, relatively few studies have explored how migrants themselves conceptualize health and how employment, legal status, social support, and healthcare interact throughout the migration process in non-metropolitan settings.

Spain offers a relevant context for addressing this knowledge gap. International migration has increasingly contributed to the demographic and socioeconomic dynamics of small towns and other non-metropolitan areas affected by aging and population decline, where migrant populations play a significant role in local labor markets and community sustainability [20]. Recent data from Spain indicate that migration contributes to demographic renewal in smaller municipalities [21,22]. In Castile and León, the foreign-born population accounted for approximately 9% of the total population in 2023 [23,24]. Provinces such as Zamora and León—classified by the OECD as TL3 non-metropolitan regions linked to small and medium-sized cities [15]—combine demographic decline with rising emigration; furthermore, they may offer fewer specialized services for migrants and less extensive support networks compared to major urban centers [25]. Research conducted in Castile and León has shown that non-urban areas can serve as locations for long-term settlement, employment, and migration projects, rather than merely acting as transit points [13,14]. Therefore, understanding the health experiences of migrants in these settings is crucial for designing equitable, context-specific health services.

The reception of migrants in Spain has also been shaped by significant changes in migration and health policies over the last two decades. Restrictive measures introduced via Royal Decree-Law 16/2012 [26] limited access to healthcare for many migrants with irregular administrative status, whereas the subsequent Royal Decree-Law 7/2018 [27] restored broader access to the National Health System, regardless of migration status. However, these legislative changes have not translated into uniform access in practice; administrative requirements, disparities between autonomous communities, and difficulties navigating the healthcare system continue to affect undocumented migrants [28,29].

Addressing these challenges is consistent with the United Nations 2030 Agenda, particularly SDGs 3 (Good Health and Well-being), 8 (Decent Work and Economic Growth), 10 (Reduced Inequalities), and 11 (Sustainable Cities and Communities), which promote equitable healthcare, social inclusion, and the reduction in health disparities [4].

This study aimed to explore how migrants living in non-metropolitan areas of Spain experience health and healthcare throughout the integration process and to identify the implications of these experiences for nursing practice, health equity, and health service responsiveness. Qualitative research is particularly suited to capturing migrants’ lived experiences and subjective understandings of health [30]. We expected participants’ narratives to demonstrate that health experiences extend beyond healthcare access and are shaped by the interaction of employment, legal status, social support, and healthcare systems.

## 2. Materials and Methods

### 2.1. Study Design

We employed a qualitative phenomenological design to explore how migrants experience health, healthcare, employment, social relationships, and adaptation throughout the migration process in a non-metropolitan context. A phenomenological approach was selected because the study sought to understand participants’ lived experiences and the meanings they attributed to health and their everyday circumstances. The interview data were analyzed using thematic analysis following Braun and Clarke [31,32]. Thematic analysis was selected because it provides a theoretically flexible and systematic approach for identifying, analyzing, and reporting patterns of meaning across a qualitative dataset [31]. This approach was considered appropriate for the present study because it enabled the researchers to examine common and divergent patterns across participants’ accounts while retaining the experiential and contextual dimensions of their narratives. The analysis was conducted iteratively, with continuous movement between the interview transcripts, coded extracts, developing themes, and the overall dataset.

### 2.2. Sample and Setting

The sample comprised 22 participants (16 women, 6 men) residing in Spain. Inclusion criteria included being aged >18 years; having lived in Spain for >1 year in a non-metropolitan area and being able to communicate in Spanish sufficiently to participate in an individual, in-depth interview without language assistance. Participants who required an interpreter to participate in the interview were excluded, as the study aimed to explore participants lived experiences through direct interaction between the researchers and participants and to minimize the potential influence of third-party interpretation on the interview data. Recruitment utilized a snowball sampling method, facilitated through face-to-face meetings with representatives from non-governmental organizations collaborating with migrants (Caritas Diocesana, Cruz Roja) [33], and two key immigrant participants who served as gatekeepers. Recruitment continued until thematic and experiential sufficiency was achieved, meaning that no substantially new dimensions of the phenomenon emerged during subsequent interviews. No participants refused participation or dropped out of the study.

### 2.3. Data Collection

Participants were contacted by phone, and upon confirming their acceptance, an individual appointment for the in-person interview was scheduled. Semi-structured in-depth interviews (the 22 interviews ranged from 23.23 to 81.25 min, with a mean duration of 46.62 min) with a guide provided by the author (available as Supplementary Material S1), were conducted by the lead researcher (CVB) between November 2023 and June 2024. The research team consisted of three nursing professionals with experience in transcultural nursing and qualitative methodology. Interviews were conducted in private settings (NGO offices or participants’ homes), with only the participant present, and were digital audio-recorded while field notes were taken for triangulation. The interview ended with a specific question about the role of the nurse. All interviews were conducted in Spanish, and no interpreter was required.

### 2.4. Confidentiality and Data Protection

Participants were informed about the confidential and voluntary nature of their participation and provided informed consent before the interview. Each participant was assigned a study code (P1–P22), which was used throughout the transcripts, analysis, and reporting of findings. Identifying information was removed from the transcripts and quotations to protect participants’ confidentiality. Audio recordings were transferred to and stored on a personal computer protected by password. Access to the research data was restricted to the research team. Data was handled in accordance with applicable data protection and confidentiality requirements.

### 2.5. Data Analysis

Data management was supported by QSR NVivo 12 (QSR International, Melbourne, Australia) to facilitate the organization, coding, retrieval, and comparison of qualitative data across interviews. The interviews were transcribed using Microsoft ®Word for Microsoft 365 MSO (Version 2608, Build 16.0.20326.20072), 64-bit, and the transcripts were reviewed by the principal investigator (CVB) to ensure their accuracy and correspondence with the original audio recordings. The interview data were analyzed using thematic analysis following the six-phase framework proposed by Braun and Clarke [31,32].

First, the researchers familiarized themselves with the dataset through repeated reading of the interview transcripts and the initial field notes. During this phase, preliminary observations and potential patterns of meaning were recorded. Second, initial codes were generated across the dataset, remaining closely grounded in participants’ accounts. Third, related codes were collated and examined to develop candidate themes and subthemes. Fourth, candidate themes were reviewed in relation to the coded extracts and the complete dataset to assess their internal coherence, distinctiveness, and relevance to the research question. Fifth, the themes were defined and named, clarifying the central organizing concept and the relationships among their constituent subthemes. Finally, the thematic structure was refined into an analytical narrative supported by illustrative verbatim extracts.

The analysis was iterative and recursive rather than strictly linear, with the researchers moving back and forth between the complete dataset, coded extracts, and developing themes throughout the analytical process [31]. Codes and themes were developed by examining patterns of meaning across participants’ accounts. As an example of the analysis procedure, an extract of the analysis, with the final set of themes, subthemes and codes, is shown in Table 1.

### 2.6. Rigor and Ethics

The study adhered to the Consolidated Criteria for Reporting Qualitative Research (COREQ) guidelines [34] to enhance transparency and methodological rigor. Throughout the research process, the investigators engaged in continuous reflexivity and phenomenological bracketing in accordance with the principle of epoché. Before and during data collection and analysis, the researchers consciously examined their pre-existing assumptions and professional perspectives regarding migration, health, employment, cultural adaptation, and healthcare. These assumptions were not considered to be completely eliminable; rather, they were explicitly identified and temporarily bracketed to the extent possible so that attention could remain focused on participants’ descriptions of their lived experiences. Reflexive field notes and analytic memos were systematically maintained during data collection and analysis to document and critically examine these preconceptions. During the analytical process, the researchers repeatedly returned to the original interview accounts and meaning coded extracts to ensure that developing interpretations remained grounded in participants’ descriptions rather than being imposed from predefined theoretical or clinical assumptions. The research team consisted of three Spanish female nursing researchers with formal expertise in transcultural nursing and qualitative methodology. While this shared professional background provided valuable clinical and contextual insight, the team remained aware of the potential risk of ethnocentric or over-medicalized interpretations. These potential influences were discussed throughout the analytical process, and investigator triangulation and consensus discussions were used to critically examine individual interpretations and refine the thematic structure.

To minimize interpretive bias, reflexive field notes and analytic memos were systematically maintained during both data collection and analysis. In addition, all interview transcripts were returned to participants for member checking, and all participants confirmed the accuracy and consistency of their statements.

Methodological rigor was further strengthened through investigator triangulation. All transcripts were independently reviewed by the three authors (CVB, EQS, EAD), and the identification of themes, subthemes and codes was achieved through a collaborative consensus process. This iterative analytical dialogue functioned as a corrective mechanism, ensuring that the final interpretive framework remained grounded in participants’ lived experiences and verbatim accounts rather than in the researchers’ pre-existing assumptions.

The study received ethical approval from the University of León Ethics Committee (ETICA-ULE-029-2023). All participants provided written informed consent prior to participation.

## 3. Results

The participants formed a heterogeneous profile with origins in Latin America (Peru, Bolivia, Ecuador, Colombia, Venezuela, Cuba), Romania, Morocco, Senegal, and Guinea Conakry (Table 2). Most were employed in precarious sectors, including caregiving, cleaning, and hospitality.

The analysis revealed that migrants’ health experiences in non-metropolitan settings were deeply shaped by structural vulnerability, particularly through the interaction between labor precariousness, administrative insecurity, social integration, and access to institutional support networks. Health was frequently perceived not as an independent state of well-being, but as a necessary condition for economic survival and social stability.

The thematic analysis identified five interconnected themes capturing the main patterns of meaning across participants’ accounts, encompassing 16 subthemes. These themes were interpreted as interconnected dimensions of structural vulnerability shaping migrants’ health experiences in non-metropolitan settings. Rather than representing independent topics, the themes describe how migration trajectories, health perceptions, social relationships, employment conditions, and adaptation interacted throughout participants’ experiences. An additional theme concerning the nursing role was analyzed separately because it arose from a specific closing question included in the interview guide (Table S1).

### 3.1. Migration Motives and Migration Processes

Migration trajectories constituted the contextual foundation of structural vulnerability. Participants’ reasons for migration, initial support networks, and subsequent administrative uncertainty shaped the conditions under which health and social integration were experienced.

*“In Peru, they started extorting and threatening us with our child. That’s why we decided to emigrate”*

(P16)

*“We’ve just got our work permit now, it was activated on the 30th, but the legal situation is still in process.”*

(15 months in Spain) (P12)

These early migration experiences established the context in which participants subsequently interpreted their health. Administrative uncertainty and the challenges of settling in a new environment shaped not only their living conditions but also the way they understood health and well-being throughout the migration process.

### 3.2. Health Perceptions (“Health as Work”)

Health was experienced and understood in relation to participants’ capacity to work and maintain economic stability. The “Health as Work” pattern of meaning illustrates how structural vulnerability became embodied in everyday understandings of health, linking physical and psychological well-being to the ability to remain economically active. 

Within the “Concept of Health” subtheme, health was consistently equated with the capacity to work (“Health as Work”):

*“For me, being healthy means being fine, being able to work, not having any issues that prevent me from doing my job.”*

(P5)

*“To be healthy means to have a job.”*

(P2)

The “Initial Emotional and Psychological Impact of Migration” was characterized by mixed experiences. Although participants valued free access to healthcare, the first months of migration were frequently associated with stress, uncertainty, and family separation.

*“I’m missing something… besides not having my daughter and not having a job.”*

(P11)

Regarding “Interactions with the Healthcare System,” experiences were generally positive, with only isolated reports of mistreatment. However, participants described psychological distress during the early stages of migration, which later shifted towards work- and family-related concerns. Within the “Health and Work” subcategory, unstable employment, unemployment, and demanding working conditions emerged as major determinants of health.

*“You’ve studied to find a job, but then you knock on doors, and none opens. So, you get depressed.”*

(P5)

*“That headache was caused by working night shifts.”*

(P14)

As participants reflected on the impact of occupational demands and emotional distress, it became evident that their capacity to cope with these challenges depended largely on the availability of social support, leading to the emergence of social networks as a key factor influencing both health experiences and integration.

### 3.3. Social Networks

Social relationships and institutional support functioned as important resources for negotiating structural vulnerability. While family and community networks could provide emotional and material assistance, limited or disrupted networks increased participants’ reliance on NGOs and other institutional sources of support.

*“Cáritas helped us a lot, especially with food and clothing… My children get help from the school canteen*”

(P15)

“Difficulties with the social network” some opposing opinions are reflected both with the immediate family, in which gender-based violence from the partner could be observed in some cases, and with their fellow countrymen, who appeared as a source of conflict.

*“No, because usually, the emigrant community doesn’t support each other. I mean, that’s a general truth. Anyone who tells you otherwise is lying.”*

(P14)

Although institutional and interpersonal support could partially buffer the difficulties associated with migration, participants consistently emphasized that employment conditions ultimately determined their opportunities for stability, autonomy, and well-being. Consequently, work emerged as one of the principal expressions of structural vulnerability.

### 3.4. Work Conditions

Employment conditions represented a major pathway through which structural vulnerability affected health. Precarious employment, excessive working hours, insecurity, and economic instability constrained participants’ ability to protect their physical and psychological well-being. 

Within the “Discrimination at Work” subtheme, discrimination based on migrant status was reported by only a few participants, although some experiences were particularly severe.

*“He threatened to send me back to Romania in a pine box.”*

(P1)

Regarding “Gender Discrimination,” participants expressed contrasting views. Some men perceived women as having greater employment opportunities due to the high demand for caregiving roles, whereas women emphasized the difficulties of work–life balance and limited access to occupations beyond domestic and care work.

*“At that time, I didn’t see it, but later… I think there’s a little more favoritism towards women.”*

(P5, male)

The “Working Conditions” subtheme highlighted unstable employment and exploitative labor practices as major barriers to health and integration.

*“I used to work fifteen hours, fourteen hours, or even more. I’d go from one place to another until 3 or 4 AM.”*

(P20)

Finally, the “Economic and Job Stability” reflected participants’ aspirations for secure employment as a pathway to legal regularization, financial independence, and access to stable housing. 

The consequences of occupational vulnerability extended beyond economic insecurity. Difficulties in obtaining stable employment and professional recognition influenced participants’ sense of identity, belonging, and long-term integration within the host society.

### 3.5. Adaptation and Integration

Integration and adaptation reflected the cumulative consequences of the preceding dimensions. Participants described difficulties in reconstructing their social and professional identities, establishing a sense of belonging, and navigating institutional expectations, illustrating how prolonged structural vulnerability could extend beyond employment and healthcare into broader experiences of social participation and identity.

Within the “Cultural Adaptation” subcategory, learning the language, adapting to the climate, and becoming familiar with cultural norms were considered essential for successful integration. The “Work and Professional Integration” subcategory reflected the need to accept jobs below participants’ qualifications or to pursue new training opportunities.

*“I haven’t had my studies validated yet… but at least I’ve done some courses, insurance agent courses.”*

(P18)

These experiences often generated frustration and diminished self-esteem.

*“I’m still nobody here. It’s like I don’t even exist in the system.”*

(P14)

Regarding “Coping Strategies,” participants described resilience, perseverance, and hope for future improvement as their main resources for adaptation.

*“I’ve been strong. Yes, without fear. I knew I had to make it happen.”*

(P2)

The “Integration Depending on Gender” subcategory highlighted additional barriers faced by women, including gender stereotypes, caregiving responsibilities, and experiences of abuse, which further complicated their integration process.

As participants reflected on these challenges, they identified nurses as key facilitators of integration, providing guidance, information, and emotional support during the early stages of settlement.

### 3.6. The Nursing Role

In addition to the phenomenological findings, participants were specifically asked at the end of each interview about their perceptions of nurses’ roles in supporting migrants. Although this category was generated from a targeted interview question rather than emerging inductively from the core analysis, participants consistently identified nurses as trusted professionals who facilitate orientation within an unfamiliar healthcare system. Nurses were perceived as key sources of information, guidance, and emotional support during the early stages of settlement, acting as key facilitators of healthcare navigation and culturally responsive care.

*“Because here you don’t know anything. You’re lost about everything. And if you don’t have someone who is there, willing to even just explain things to you… you’ve just arrived.”*

(P14)

Overall, the findings support an interpretive model (Figure 1) in which migrants’ health experiences are shaped by cumulative structural vulnerability. Migration trajectories initiate a process of administrative precarity that contributes to occupational vulnerability, influences perceptions of health as the capacity to work, and ultimately affects identity, social integration, and healthcare experiences. Social support, particularly from third-sector organizations and nursing professionals, emerged as an important protective factor capable of mitigating these structural challenges. 

## 4. Discussion

This study describes how migrants living in non-metropolitan Spanish cities experience health, primarily through the lens of structural vulnerability. Health was closely associated with the ability to work, maintain legal stability, and sustain family responsibilities, revealing how labor precariousness and administrative insecurity function as major social determinants of health. Consistent with previous studies [17,35], migrants’ health experiences were also linked to their perceived social acceptance and integration within the host society.

Unlike large metropolitan areas, non-metropolitan settings may offer fewer informal migrant networks and less culturally adapted institutional infrastructure, increasing dependence on third-sector organizations for social and healthcare navigation. While migration research has traditionally focused on cities such as Madrid or Barcelona, this study contextualizes migrant health within smaller urban centers such as Zamora and León, which face distinct socioeconomic pressures related to demographic aging and population decline. Participants frequently described migrant networks as fragmented or limited rather than consistently protective, increasing feelings of uncertainty and social isolation during the integration process.

Consequently, formal institutional networks, particularly organizations such as Cáritas Diocesana and the Red Cross, emerged as essential mechanisms for social and structural integration. In these contexts, NGOs not only provided material assistance but also functioned as key mediators for institutional orientation, healthcare navigation, and psychosocial stability during the early stages of migration.

One of the principal findings of this study, the concept of “Health as Work”, captures how participants understood health primarily in relation to their capacity to work and maintain economic stability. Rather than being perceived as an independent state of well-being, health was experienced as a prerequisite for economic survival, while occupational vulnerability, unstable working conditions, and irregular administrative status contributed to psychological distress and insecurity. These findings are consistent with previous research identifying immigration status, employment conditions, and socioeconomic insecurity as important determinants of migrant health [35,36]. Similar patterns have been reported in other contexts: a qualitative study in Colombia found that precarious employment affected immigrants’ physical, mental, and psychosocial health, while also limiting access to healthcare and labour rights [18,37], whereas qualitative research in rural Australia highlighted the role of employment opportunities, social connectedness, discrimination, and access to services in migrants’ health, well-being, and integration. However, our findings extend this perspective by showing how these interconnected structural disadvantages were experienced throughout the migration process and incorporated into participants’ own understanding of health. Thus, “Health as Work” provides an experiential expression of structural vulnerability, illustrating how broader social and economic conditions can shape what being healthy means in everyday life.

Mental health difficulties evolved throughout the migration process. Initial stress and anxiety related to adaptation gradually shifted toward distress associated with work instability, family separation, and social relationships, consistent with previous research [38]. Although most participants reported generally positive interactions with healthcare professionals, limited institutional familiarity and administrative uncertainty often complicated healthcare navigation. Access to public healthcare was perceived as especially important, particularly within the framework of Royal Decree-Law 7/2018 [27], which guarantees healthcare access regardless of migratory status in Spain.

These findings reinforce the need for nursing professionals to move beyond exclusively biomedical approaches and incorporate structurally informed and culturally responsive perspectives into migrant care. Participants emphasized the importance of empathy, active listening, clear information, and support in navigating unfamiliar healthcare systems, in line with international recommendations for culturally competent nursing practice [39,40]. In non-metropolitan areas, where nurses may encounter migrant populations less frequently than in large urban centers, cultural competence training and reflective practice become particularly important [41,42].

These findings highlight the importance of considering the broader social and structural conditions shaping migrants lived experiences of health in non-metropolitan settings. The heterogeneity in participants’ length of residence provides an important context for interpreting these findings. Experiences of healthcare navigation, social support, and integration may evolve as migrants become more familiar with institutional procedures and develop social and professional networks, consistent with previous qualitative research highlighting the role of social connectedness and integration in migrants’ health and well-being [18,36]. The findings should be understood as reflecting diverse experiences across different stages of settlement rather than as evidence of a specific temporal trajectory.

Although the study was conducted in only two provinces, its findings may provide transferable insights for other non-metropolitan areas of Spain that share similar demographic, socioeconomic, and migration-related characteristics. However, transferability should be considered in relation to the specific territorial and healthcare context of each region. In Spain, primary healthcare is managed largely at the level of the autonomous communities, which have substantial responsibility for healthcare planning, resource allocation, and service provision. Therefore, regional differences in healthcare organization and resources may influence migrants’ experiences. The findings should consequently be interpreted as context-specific experiences that may inform understanding and further research in comparable non-metropolitan settings rather than as representative of migrant populations across Spain.

### Study Limitations

This study reflects the experiences of migrants recruited through two NGOs in Zamora and León; therefore, it does not aim to provide representative evidence for migrant populations across Spain. The transferability of the findings may be influenced by regional differences in demographic characteristics, migration patterns, social resources, and the organization of healthcare services across autonomous communities. Nevertheless, the study provides context-specific insights that may be relevant to other non-metropolitan settings with comparable characteristics. Participants varied substantially in their length of residence in Spain, which may have influenced their familiarity with healthcare services, institutional procedures, social networks, and integration processes. Although this heterogeneity was considered during interpretation, the study was not designed to compare experiences according to length of residence. Additionally, the requirement for sufficient Spanish proficiency and the absence of interpreter support may have limited the representation of participants with limited Spanish proficiency. Furthermore, participants’ precarious work schedules limited opportunities for data collection. 

## 5. Conclusions

This study suggests that migrants living in non-metropolitan Spain experience health through the interaction of structural vulnerability, labor conditions, administrative status, and social integration. The concept of Health as Work illustrates how health is primarily valued as the capacity to sustain employment and economic stability rather than as a state of well-being. These findings show that migrant health cannot be understood independently of the structural conditions that shape access to employment, healthcare, and social participation.

Comparative studies and intervention research are needed to evaluate how culturally responsive and structurally informed healthcare, including nursing interventions, can reduce structural vulnerability and improve migrant health equity.

## Figures and Tables

**Figure 1:**
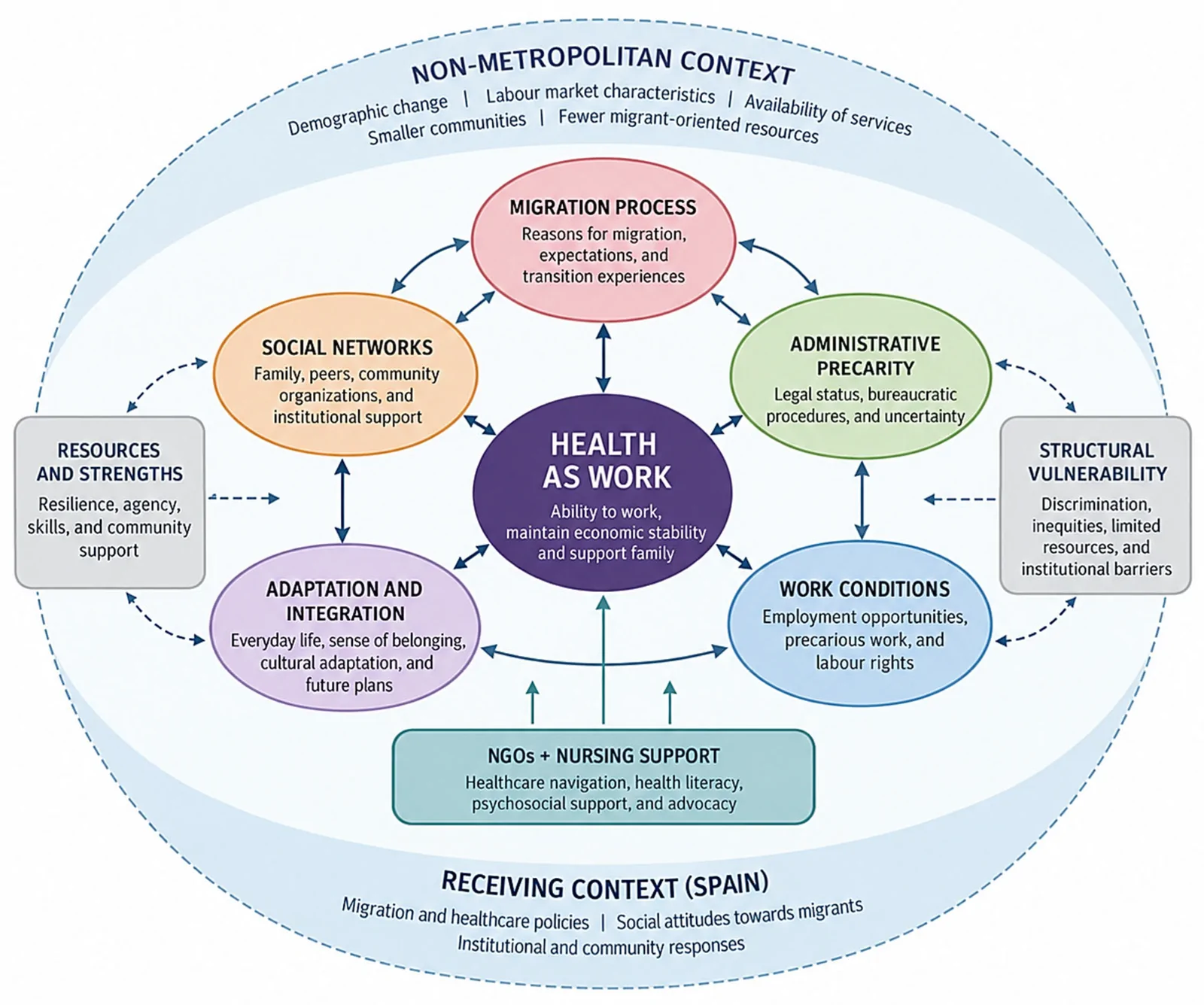
Interpretive representation of the interconnected dimensions shaping migrants’ health experiences in non-metropolitan Spain. The figure provides an interpretive representation of the relationships identified across participants’ accounts and does not imply linear or causal relationships. “Health as Work” is positioned as a central interpretive dimension, reflecting how participants understood and experienced health in relation to their capacity to work and maintain their livelihoods. “Structural Vulnerability” and “Resources and Strengths” are positioned as lateral dimensions that shape this experience. Migration trajectories, administrative conditions, work conditions, social networks, and adaptation and integration are represented as interconnected dimensions through which these influences are manifested. Nursing support is represented as a supportive resource within this context. Solid lines indicate direct and reciprocal relationships between the interconnected dimensions. Dashed lines indicate contextual or supportive relationships that may influence or mitigate these dimensions, without implying a direct or causal pathway.

**Table 1:** Example of Representative Themes and Verbatims.

Themes	Subthemes	Illustrative Verbatim
Migration Motives	Security	“In Peru, they started extorting and threatening us with our child. That’s why we decided to emigrate.” (P16)
Health Perceptions	Health as Work	“To be healthy means to have a job… without health you have nothing, no matter how good the job is.” (P5)
Social Networks	Institutional Support	“Caritas helped us a lot, especially with food and clothing… My children get help from the school canteen.” (P15)
Work Conditions	Precariousness	“I used to work fifteen hours, fourteen hours, or even more. I’d go from one place to another until 3 or 4 AM.” (P20)
Adaptation	Loss of Identity	“I’m still nobody here. I don’t even exist in the system.” (P14)

**Table 2:** Sociodemographic Profile of Participants.

Code	Gender	Country of Origin	Age	Years in Spain	Education Level	Legal Situation
P1	F	Romania	40	17 y	University	Legalized
P2	F	Romania	46	21 y	High school	Legalized
P3	F	Bolivia	33	14 m	University	Legalized
P4	F	Ecuador	52	21 y	University	Legalized
P5	M	Ecuador	52	18 y	High school	Legalized
P6	F	Cuba	51	23 y	University	Legalized
P7	F	Romania	47	17 y	High school	Legalized
P8	F	Paraguay	55	18 y	High school	Legalized
P9	F	Peru	48	14 y	High school	Legalized
P10	F	Cuba	46	23 m	High school	Legalized
P11	F	Peru	46	17 m	High school	Irregular/in process
P12	F	Peru	36	14 m	High school	Irregular/in process
P13	F	Dominican Republic	39	11 y	University	Legalized
P14	F	Venezuela	33	18 m	University	Irregular/in process
P15	F	Colombia	48	4.5 y	High school	Process revision
P16	F	Peru	31	15 m	High school	Irregular/in process
P17	F	Morocco	34	15 y	High school	Legalized
P18	M	Venezuela	41	3 y	University	Irregular/in process
P19	M	Peru	42	2.5 y	High school	Asylum/in process
P20	M	Morocco	35	7.5 y	High school	irregular/in process
P21	M	Guinea	24	2.4 y	Basic	Irregular/in process
P22	M	Senegal	24	2 y	Basic	Irregular/in process

## Data Availability

Due to the sensitive and personal nature of the qualitative interviews, the participants did not consent to their full transcripts being made publicly available. Consequently, the data are restricted to protect participant confidentiality and comply with ethical guidelines. Anonymized excerpts may be available from the corresponding author upon reasonable request.

## Supplementary Materials

The following supporting information can be downloaded at https://www.mdpi.com/article/10.3390/healthcare14193202/s1. Supplementary Material S1: Semi-Structured Interview Guide. Table S1: Categories, subcategories and codes.
