# Supplementary material for: “Health as Work”, Structural Vulnerability Shaping Migrant Health in Non-Metropolitan Spain: A Qualitative Study
Source: Healthcare (Basel). 2026 Sep 28;14(19):3202. doi: 10.3390/healthcare14193202 (PMC13665365; doi:10.3390/healthcare14193202)
Supplement: Supplementary file 1 [file healthcare-14-03202-s001.zip › healthcare-4514321-supplementary.pdf]

SUPPLEMENTARY MATERIALS

Supplementary Material S1. Semi-Structured Interview Guide

Interview Number: _______

Participant Code: _______

Before the interview: Introduction. Reading and signing the informed consent form.

Sociodemographic Information

- Country of origin (country, geographical region, linguistic area);
- Age;
- Sex;
- Marital status;
- Number of children;
- Length of residence in Spain;
- Language proficiency / Native language;
- Educational level in the country of origin.

Educational level in Spain Literacy (reading, writing, basic numeracy)

- Professional qualifications and vocational training;
- Professional and employment experience in the country of origin (type of employment, job position, professional activity, salary, employment stability, social benefits);
- Professional and employment experience in Spain (type of employment, job position, professional activity, salary, employment stability, social benefits);
- Professional and employment experience in other countries (type of employment, job position, professional activity, salary, employment stability, social benefits).

Legal and administrative status

Religion or religious affiliation (optional)

Interview Questions

Health and Migration

1. How did you decide to migrate? Did anyone help you? (Personal and family goals, migration strategies.) What were the main difficulties you experienced?

2. What does be healthy mean to you? How has the migration process affected your health?

3. How have you felt since arriving in Spain? Do you think any aspect of your health has changed? If so, what has changed? What do you think caused this change?

4. How are you feeling emotionally? What are the main difficulties you face in achieving well-being or happiness here?

5. What has your experience been like in seeking healthcare or medical treatment in Spain? Have you found it easy to access healthcare or emotional support (primary

healthcare centers, hospitals, mental health services, etc.)? Would you like to share any particular experience?

6. Are you currently employed? How did you find your job? What is your work like? (Working hours and working conditions.)

7. How would you describe your treatment at work? Have you experienced discrimination? (Relationship with employers and colleagues.)

8. Do you think being a woman/man has benefited or disadvantaged you compared with other migrant men/women? (Perceived gender differences.)

9. Do you have family members in Spain? Do you live with them? What is your relationship to them?

10. Have you developed close relationships with people from your own culture or from other cultures within your community? How have these relationships influenced your acculturation process and emotional well-being?

11. Are you involved in any organization or association? What has your experience been? (Associations of people from your country of origin, community meeting places, embassies, consulates, organizations providing support, reception and counselling services, social services, local institutions.) Have these organizations helped you establish new informal connections? How have these interactions influenced your acculturation and integration process?

12. In your opinion, how could nurses help improve your situation?

Table S1. Categories, subcategories and codes

(For readability, Supplementary Table S1 is presented in landscape format.)

Themes	Subthemes	Codes	
**Motives and Migration Processes**	Motives	Economic situation	P2: "I wanted a better life because in my country they paid very little, it wasn't enough to make a living and of course, it's the dream of all young people to want to have a better life."<br>P6: " The economic situation in Cuba, and also the way I am… they would have put me in jail because I worked… I wrote poems, I did things and one day they would have put me in jail and there would be no coming back."<br>P7: "Due to a lack of money and definitely due to a lack of work. Yes, well, one of my sisters helped me. Emm, to come only to find a job."<br>P4: "The motivation was because back in our country, you know, I was in a bad situation. My children in my house, my home. So, I said, no, I have to leave, the only solution is to emigrate here to be able to raise my children.
		New Opportunities	P5: "At first, out of curiosity. Because I have a friend who has been here for about twenty-eight years and he, well, every time he came, he would tell us about how things were. And of course, first of all, you see the economic situation there, the work situation, that is the difference.<br>P1: "My biggest motivation was the backward mentality of my country, that is, how to evolve or how to move forward. I did not share their way of thinking at all."
		Personal and Family Reasons	P9: "Fernando and I met, as they say, at work. He had a travel agency before... We went to cater Peruvian cuisine, and we exchanged business cards... So, when I came here, I was already married because he came back with a ring in his hand."<br>P3: "Basically it was meeting my husband, my current husband is Spanish, and I believed, I mean, I thought that I would have, I mean, a new opportunity in a new country."
		Harassment/ Security	P8: "My motivation was... my partner was jealous and an abuser. So… I escaped from him and came here, very well, because my sister was already here."<br>P15: "We arrived here with political asylum.../ My husband was travelling all over Colombia and he was going to Ecuador. They started threatening... Before I knew it, I had already had to quit working... so we came because there was a lot of insecurity and constant threats."<br>P16: "In Peru, they started extorting us and threatening us with our son. That's why we decided to emigrate."

	Migration Process	Initial Contact Network	P4: "I contacted some people there who were from the same city and who were already here. They helped me get here."<br>P1: "It was my own decision. I wanted to go to the United States and migrate to the United States. I didn't get the visas and... and nothing. So, I resigned, I signed off work and I said I'm going to Spain."<br>P5: " Yes, I have a friend who is in Malaga and as I told you, he has been here for about twenty-eight years."
		Administrative Processes and Obstacles	P9: Yes, yes. No, in that sense yes, but I have to have it in my papers. And well, I gave it up. I said, well, no… there are other expenses, but now, so to speak, I always put it off, I put it off and that's it.<br>P12: "We've just got our work permit now, it was activated on the 30th, but the legal situation is still in process."<br>P13: "I started the process. First, I had to validate my high school diploma and then start the university process... it took a long time, and there are a lot of people who give up because of course they ask you so many things and put so many obstacles..."
**Health Perceptions/Health Experiences**	Concept of Health	Integral Concept of Health	P1: "Health is not only physical, but also mental. There is a Latin proverb that says: A healthy mind in a healthy body."<br>P2: "To be healthy, healthy is to have peace, achieve what you have wanted."<br>P3: "... Cover... I mean, having all my physical and emotional needs met, so to speak. Well, no, not feeling any pain, at all."
		Health is Work	P2: "To be healthy is to have a job."<br>P4: "Health is essential for us, for people, because if we're not healthy, we cannot work."<br>P5: "For me, being healthy means being fine, being able to work, I don't know, not having any issues that prevent me from doing my job."
	Initial Emotional and Psychological Impact of Migration	Free Healthcare	P5…because health is not like here... it is free, but you have to wait a long time before you are taken care of. So, it is a little risky to be ill over there... because who knows when you will be assisted, and here it is different."<br>P16: "the advantages that Spain had over my country, because in my country health costs money and here it doesn't. Health is public and free for everyone, without, how do they say it, without discriminating against anyone due to economic circumstances."

		First Impact and Stress	P2: "... I didn't even know. I'd heard of Spain, but I didn't know what Spain was like. When I got off the bus, it was all different from what I'd experienced. And I was stunned. I said, what have I done? Where am I?"<br>P4: "When you first arrive as an immigrant, the situation is difficult, really difficult. First, because we come to another country where we don't know anyone, and we don't have papers. We can't get sick, we don't have a way to go to the doctor."
	Interactions with the Healthcare System	Relationship with Healthcare Professionals (support)	P2: "They always called me. How are you? They come and visit you. The midwife. Very good. Everyone fantastic, fantastic."<br>P3: "The family doctor, the truth is, very good, as I understand it, very fast,"<br>P11: Well, they told me that what happens here is that they don't give you what... in my country you go to the pharmacy and they give you a vial or they give you something for the fever. However, here just take paracetamol and then Ibuprofen, I mean, it seems to me that those are the medications they give you here."
		Relationship with Healthcare Professionals (mistreatment)	P8: "I have a burn covering forty-five percent of my body and, well, part of my abdomen and right thigh and part of my left thigh where they did the graft. That's when I realized that some women were very good and others treated me badly. I think it's because I'm an immigrant."
	Mental Health	Mental Health and Depression	P1: "I went through a very big depression. I dragged around seven years of depression without knowing it until one day I saw it."
		Family Problems	P6: "Right now I have a lot of problems, a lot of problems in the sense that, but well, everyone sees their own problem as they can, but there are people who are worse off and you have to be thankful and many things, because if I was… when my husband died a month ago, I was…"
	Health and Work	Impact of Work on Health	P2: "One thing I misunderstood is that I started working, working, working. And I didn't realize that when something starts to hurt, I ignored it, I said 'no problem, I'll take a paracetamol, I'll take a nolotil, and I have to work harder.' It's a trap, you see? And that's when I realized that you think you're achieving something... and you're not."<br>P14: "I was recently admitted to the hospital for a headache that left me almost unconscious. But that headache was the result of working nightshifts in Siro, in the mornings taking the child to school, the youngest one has to have his adenoids and tonsils operated... / I mean, you have to stop or I'll make you stop, literally. My body made me stop. I mean, my body stopped me. No, that's it, up to here, very well."

**Social Relationships and Support**	Social Networks Support	Family and Community Support	P3: "Yes, well, my family supported me. Whatever decision I made, they were going to support me. But of course, my husband was the one I was most in contact with because we had to figure out the legal issue."<br>P5: "Thanks to many friends I have here or acquaintances that you find along the way, and they help you out."<br>P7: "This family (the first one they worked for) helped me bring my husband and give him a work contract so that I could rent a flat."
		Institutional Support	P2: "I went to Caritas, and they did take notes to see if they could find a woman I could look after…"<br>P3: "No. Well, I volunteer for the Red Cross, but I go as a volunteer."<br>P8: "Yes, I have gone to Caritas, which helped me, and to the Red Cross as well. For a while./<br>P8: "I am in Comisiones Obreras, I am on their job placement list.
		Associations	P5: "Yes, that helps you a lot. Being surrounded by a lot of people makes you, as you said, more sociable. Then you interact with people from one office, from another with… with public servants and you, I mean, it opens up more paths for you. I mean, maybe not regarding work, but it does enrich you more in social knowledge, in getting along with people, in helping, in whatever. I mean, it makes you feel more useful. Yes, yes. It is very, very, very gratifying to be involved
	Difficulties with Social Network	Immediate Family and Gender Violence	P17: "Well, before, as I told you, if he didn't, if he didn't go with me (her husband), I wouldn't go to the doctor and when he's with me you're afraid to talk, you know, about what really happened to you. What's really happening to you? Well, you don't tell the truth."
		Compatriots	P7: "For example, I had problems with my compatriots. I mean, they brought me here to Zamora, they helped me and then they started to get really hard on me for money for the room they gave me and I didn't know anyone. I knew them and the people of the house they brought me in to work. In this house, they didn't understand me very well, what I was saying, and they took advantage of me, they told me that they were the ones paying me. I mean, it was like a business, almost, .../ Well, I got to the point where my countryman controlled me, he controlled my money, he paid me whatever he wanted."
**Employment Situation**	Discrimination at Work	Discrimination for being an Immigrant	P15: "Well, when I first arrived, I had to look after some old ladies. I understand that when you get older, dementia and all that stuff starts, right? One tries to handle things as they are, right? But yes, I've had an altercation

			with an elderly woman who said I was black, it was funny, she told me not to touch her and whatever."
		Gender Discrimination	P3: "So I don't know if gender counts, but in general, let's say that I haven't personally experienced anything like having been discriminated against as a woman, I couldn't say."<br>P4: "That's in that regard. I think that women have a little more priority to go and take care of older people, because, for example, if the people we are going to take care of are women, the daughters prefer a woman to go and look after them, rather than a man. Well, some men, yes, there are more now. There are more male caregivers because before they were not seen much, but now they are. It seems that these caregivers are coming in to take care of older people, men I mean"
		Lack of Direct Discrimination (perceived)	P17: "Well, I lived in a building before where the neighbours, when they greet you, when they talk to you, they ask you. This also matters, right? Because I lived in another building, in another house that you... well... like they don't want you in that building. Because you're a foreigner, I don't know, a Moor as they call us or something like that. Well, they don't want you. But when you're in a building where they greet you... you know, they say hello, how are you or what's up? How are the kids
	Working Conditions	Distrust	P6: "I saw many things and I thought maybe he was going to prostitute me and all, it did get into my head. One day I hid, I got dressed and everything, and I said, when I have the chance I'm running away, I escape from him and ask where the police are."
		Endure	P9: "The clients themselves tell me 'if you weren't there, this wouldn't work'. I mean, it's something like that, you say you're indispensable to him now. In a certain way, they say it. And I say 'No, no, he's the boss here and he's the professional', he always tries... 'No, no, no, Lucía, you... You do a lot here, even if you don't believe it'. And I say no, but this isn't my thing."
		Work Difficulties	P10: "I mean, I used to work fifteen hours, fourteen hours, or even more. I'd go from one place to another until 3 or 4 AM. Well, of course."<br>P12: "Well, they paid me for that day, for that day and they told me here it is... Then they worked daily... / And then yes but well treated."<br>P13: "and I do, I mean, it's not a job per se, because it's like, for example, things like decorating or birthdays. Things like that... / But again, it can be months and especially around this time of communions and Christmas,"

			P20: “By days, you come in one day, you sign an eight-hour contract that day and the next day you finish your working hours and your contract is already over.”
	Economic and Work Stability	Work Stability	P10: “I've had my papers ever since I arrived. I got a job on the very day I got my papers. Well, look, I haven't encountered any difficulties, to be honest.”<br>P11: “Work, what work I would like, finding a job, a good job, that doesn't neglect my family either. No, because the job I have is, how to put it, enslaving.”
		Access to Housing	P12: "Things are different here because you have to work with papers, without papers... We are in the process."<br>P14: "She was the one who, in order to rent, you need to have at least one year of Social Security or an indefinite contract... My aunt was the guarantor of the flat and she was the one who paid the month's deposit and the first month."
**Integration and Adaptation**	Cultural Adaptation	Language Learning	P17: "The language also has an impact, or if I had gone out more… Because now I can speak, but if you had asked me to participate in this back in 2009, 2010, or so—no, no, no, no. But now I feel much better at socializing, and that really makes a difference. If you don’t interact with people, you just stay isolated. And that also affects your health."
		Cultural Adaptation and Climate	P13: “because here, the climate is harsher too, the climate you feel it a lot, very much, because of course, the weather there is the same now. The seasons of the year change, even on the same day.”<br>P18: “So, of course, it's a culture very similar to ours. It's not such a shock as when you go to another place where the culture is different. The accent is different, you know? It's like completely different words. Here we are very similar. Therefore, you feel like, as you're already making a change, a big change in your life, at least you're in a place where it's easier for you to settle down.”
	Work and Professional Integration	Professional Changes	P10: “I studied economics... Accounting technician.../ working in the Atentos company, (care for the elderly)”<br>P12: “The training that we have received is that of warehouse assistant. In my country, warehouse is mostly for men, but look, here they gave us this course and it is something that a woman can do, because even I got to drive, because they have already given us the license.”

		Changes in the Academic Training	P1: "As soon as I arrived, I took the university entrance course for those over twenty-five years old due to the lack of validation of my qualifications and that of nursing assistant."
		Unmet expectations	P3: "Well, anyway, that's all it is, isn't it? The fact is that I've left everything, that I had already everything done, and here, I have nothing anymore... Yes, well, basically it's starting from scratch again and not being worth much here. Yes, but well... (in tears)"<br>P18: “I haven’t had my studies validated yet, I’m waiting for the new certificates to do it, but at least I’ve done some courses, insurance agent courses."
	Coping Strategies	Resignation/resilience	P2: “I’ve been very strong. Yes, without fear, without anything. I knew I had to make it happen. And I knew that…”<br>P6: “But well, you must accept the things that you cannot change and also try and think, be empathetic to the people, the conditions they have been through, how they feel. And that is the most important.”
		Expectations of change	P14: “I mean, because you are integrated. I mean, you are not the immigrant that, I mean, you have to see how, usually you feel rejection. Well, but I realized that the rejection is not on their part. The rejection is yours. Mentally, you are the one who isolates yourself because I see it a lot. At school I see how people who arrive, you try to involve them, to get them to join, and they don't participate. I mean, they reject themselves.../I think out of fear or shame, you know that you are starting from scratch. You have many economic needs and for example, at the beginning, it happened to me.”
	Integration Depending on Gender	Abuses	P6: “Anecdotes that have happened to me: a neighbour who lives on the third floor... / He went in through the door to the outside, to the living room, and I said ‘what?’, trying to kiss me and I said ‘get out of here immediately, look, I have the alarm on...’ /”<br>P5: “Then she couldn't find work, the doors were closed to her. Then, suddenly, not finding work here, having the doors closing on you, no matter whether you go knocking on one or the other, suddenly pushes you into prostitution. And that is hard, of course, and there are people who do that.”
		Negative image of immigrant women	P20: “People have an image that the… that the, but if I have not even given motives, my way of thi… of thinking, of speaking and of acting, because look, I am quite distant in that aspect that, I’m telling you, I am not one to be…/ But with all the years that I have been here, that they’ve known me, that I am

	not one to go to a neighbour's house or to… I just greet people ‘Good morning. Good afternoon.’ I am not interested in anyone's life, anyone's.”
**The Nursing Role**	P5: "It could help a lot. Do you know why? Because there are people like I told you who get depressed, people who come, eh, leaving their children, people who come, leaving their family, and well, especially women who are very, very sensitive, they are more sensitive, eh... And well, I’d say, if you have more knowledge of how to help them and how to get in there, well, you're going to help them and... and of course, there are people who do. There was a friend who said that she was going to commit suicide because she couldn't find work, she couldn't find work, and that left a mark on us. Then she couldn't find work, the doors were closed to her. Then, suddenly, not finding work here, having the doors closing on you, no matter whether you go knocking on one or the other, suddenly pushes you into prostitution. And that is hard, of course, and there are people who do that, but there are people who put up and put up with it, and they are in this in search of a job, to work, to work by the hour. But I say that if, for example, the nursing sector or nurses, they also have a psychological impact and they can get in it. Help them like we do, as an association. Let's say, we are in it, but if we know that there is an association of nurses as well or of nurses who are dedicated, then we can send them people so they can guide them much better in that”
